# A deep learning approach to identify and segment alpha-smooth muscle actin stress fiber positive cells

**DOI:** 10.1038/s41598-021-01304-4

**Published:** 2021-11-08

**Authors:** Alexander Hillsley, Javier E. Santos, Adrianne M. Rosales

**Affiliations:** 1 McKetta Department of Chemical Engineering, University of Texas at Austin, Austin, TX USA; 2 Hildebrand Department of Petroleum and Geosystems Engineering, University of Texas at Austin, Austin, TX USA

**Keywords:** Computational models, Image processing, Machine learning, Biomaterials - cells

## Abstract

Cardiac fibrosis is a pathological process characterized by excessive tissue deposition, matrix remodeling, and tissue stiffening, which eventually leads to organ failure. On a cellular level, the development of fibrosis is associated with the activation of cardiac fibroblasts into myofibroblasts, a highly contractile and secretory phenotype. Myofibroblasts are commonly identified in vitro by the de novo assembly of alpha-smooth muscle actin stress fibers; however, there are few methods to automate stress fiber identification, which can lead to subjectivity and tedium in the process. To address this limitation, we present a computer vision model to classify and segment cells containing alpha-smooth muscle actin stress fibers into 2 classes (α-SMA SF^+^ and α-SMA SF^-^), with a high degree of accuracy (cell accuracy: 77%, F1 score 0.79). The model combines standard image processing methods with deep learning techniques to achieve semantic segmentation of the different cell phenotypes. We apply this model to cardiac fibroblasts cultured on hyaluronic acid-based hydrogels of various moduli to induce alpha-smooth muscle actin stress fiber formation. The model successfully predicts the same trends in stress fiber identification as obtained with a manual analysis. Taken together, this work demonstrates a process to automate stress fiber identification in in vitro fibrotic models, thereby increasing reproducibility in fibroblast phenotypic characterization.

## Introduction

Fibrosis is a pathological process that affects millions of Americans each year and can manifest in nearly every organ in the body, including as cirrhosis in the liver (2.5 million people^1^), idiopathic pulmonary fibrosis in the lungs (5 million^2^) and as heart failure (6 million^3^). Due to its development in multiple tissue environments, several factors can lead to fibrosis. For example, cardiac fibrosis has a range of etiologies, including acute events such as myocardial infarct^4^ and chronic conditions such as hypertension^5^ and aging^6,7^. Regardless of the tissue or initiating injury, fibrosis is characterized by excessive tissue deposition and matrix remodeling, leading to increased stiffness and impaired organ function^8,9^.

On a cellular level, fibrosis is associated with the persistent activation of fibroblasts into myofibroblasts, a smooth muscle cell-like phenotype presumed to be highly secretory^10^ and contractile^11–13^. Myofibroblasts are most often identified in vitro by the de novo assembly of alpha-smooth muscle actin (α-SMA) stress fibers^14–16^, although recent research has shown that α-SMA^+^ cells are not the only types of fibroblasts responsible for secretion and contractility during fibrosis in vivo^17,18^. Despite the fibroblast heterogeneity in vivo, numerous studies in vitro and in vivo have shown that increasing matrix stiffness is both a cause^19^ and a result^20^ of increasing α-SMA expression, creating a positive feedback loop that, when uncontrolled, progresses to pro-fibrotic cellular phenotypes. Synergistic processes such as TGF-B signaling^21,22^ and the innate immune response^23^ also lead to increased myofibroblast activation as identified by α-SMA stress fibers. Due to the organization inherent to their assembly, α-SMA stress fibers are most commonly identified via manual classification using fluorescence microscopy and immunohistochemical staining.

**Figure 1:**
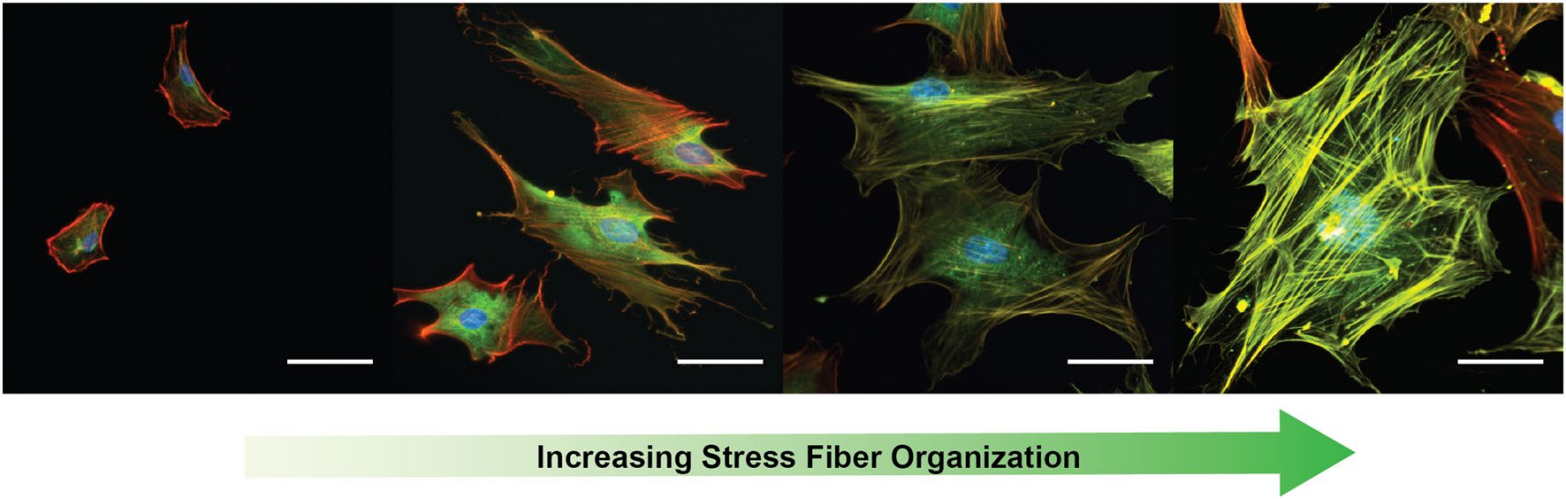
Fixed and stained cardiac fibroblasts with increasing levels of α-SMA stress fiber organization. Myofibroblast activation has been shown to correlate with α-SMA SF^+^ phenotypes. Red: F-actin, Green: alpha-smooth muscle actin, Blue: Nuclei, scale bar = 50 μm.

Although α-SMA stress fibers represent a hallmark of the myofibroblast phenotype, almost all fibroblasts express a base level of diffuse α-SMA^24^ (Fig. 1). This differential expression complicates the manual identification and classification of cells with stress fibers, leading to a time-consuming process. Manual identification also lends itself to reproducibility problems and user bias, which can lead to variations in cell classification between experiments, researchers, and labs. Previous attempts to automate this process have used methods such as flow cytometry^25^ and simple intensity measurements through packages such as FIJI^26^ to determine the average α-SMA expression of the cell population. However, these methods yield results that correlate overall α-SMA expression with staining intensity, which may overlook stress fiber organization. The organization of α-SMA into stress fibers is responsible for many of the phenotypic behaviors associated with myofibroblast activation^27^ and therefore represents a key aspect of the myofibroblast classification process^28^. To this end, an automated method of image-based cell identification that is based on fiber structure would both reduce time spent on this tedious task and increase consistency in the field. Although the degree of α-SMA stress fiber formation exists on a spectrum, it is possible to differentiate between cells that contain no stress fibers (α-SMA SF^-^) and those that contain at least a single stress fiber (α-SMA SF^+^). Therefore, we sought to train a model to classify cells based on stress fiber presence.

Automating the identification of cells containing organized α-SMA stress fibers (referred to as α-SMA SF^+^) presents a complex computer vision problem due to the following reasons: (1) fiber organization does not necessarily correlate with staining intensity, (2) all cells have unique, often sprawling shapes, and (3) most α-SMA SF^+^ cells are heterogeneous and also have regions of diffuse α-SMA. Because of these complexities, no pre-existing image processing algorithm is well suited for this task.

Convolutional neural networks (CNNs) have recently become a powerful tool for solving image segmentation problems^29^. CNNs are machine learning models that utilize multiple levels of abstraction to learn the identifying features of an image^29,30^. Previous work has combined CNN-based frameworks with high throughput flow methods to differentiate up to 4 different cancer cell lines simultaneously^31,32^, classify white blood cell types^33^, and identify various types of HEp-2 cells^34^. However, all of these methods classify images containing a single, suspended cell. Classification of images containing multiple adherent cells are less common^35^ but has been applied to identify subcellular features in publicly available datasets of HeLA^36^ and CHO^37^ cells. These previous reports are all examples of image classification, where a label is assigned to the image as a whole (i.e., all cells in the image are of the same “type”). In contrast, the classification of images with multiple distinct cell phenotypes (e.g., “α-SMA SF^+^” vs. “α-SMA SF^−^”) requires semantic segmentation, where a label is assigned to each pixel within the image^38^. Semantic segmentation is often approached using an encoder–decoder architecture, which contains 2 paths: (1) an encoder that uses a series of convolutions to learn the most essential features, and (2) a decoder that upscales those features to create a segmentation map of the original image. This type of model was first utilized by Ronnenberger et al. in their UNet^39^ and has since been built upon, including but not limited to ResUNet^40^, ENet^41^, FRRN^42^, and ERFNet^43^. This type of segmentation allows us to process more complex images (for example, one with multiple cell types) and allows for further analysis such as measuring cell shape and size. Semantic segmentation of cells is an active area of research^38,44–48^; however, most efforts have focused on the task of segmenting cells from tissue or background, rather than distinct cell types from each other.

Here, we report the design and application of a computer vision model trained to classify two different phenotypes of cardiac fibroblasts in the same image, based upon the presence of α-SMA stress fibers within each cell. Specifically, we combined traditional image processing techniques with a model based on well-established semantic segmentation components. To train and test our model, we generated heterogeneous fibroblast samples with mixed cell phenotypes using hyaluronic acid (HA) hydrogel substrates of various elastic moduli, spanning a range that mimics healthy tissue and fibrotic tissue to mechanically promote α-SMA stress fiber organization. After two, four, and six days of cell culture, we stained for α-SMA stress fibers and imaged the samples using fluorescence microscopy. We demonstrate that this model can accurately segment both α-SMA SF^+^ and α-SMA SF^−^ cells within a single image and capture changes in cell phenotype following a change in substrate stiffness. Because α-SMA stress fiber formation is common to myofibroblasts from many different organ and tissue types^49^, our model has the potential to be broadly applied to other cell types.

## Materials and methods

### Cell culture

Normal human cardiac fibroblasts (NHCFs, Lonza) were thawed from cryogenic storage at passage 5 and expanded for a single passage (4 days) on 100 mm tissue culture polystyrene petri dishes. NHCFs were expanded in complete DMEM with 10% fetal bovine serum (Corning) and 1% penicillin/streptomycin (Fisher Scientific). Cells were then removed from the plate using 0.25% trypsin solution and seeded on hydrogels in a 12 well plate at a seeding density of 3000 cells/cm^2^. For the stiff control hydrogels, the seeding density was slightly lowered to 2000 cells/cm^2^ to achieve similar confluency as the soft hydrogels at 6 days. Low seeding densities were chosen to reduce cell–cell contact when imaging. During culture on hydrogels (up to 6 days), complete media was changed every other day. All cells were cultured in an incubator at 37 °C containing 5% CO_2_.

### Methacrylated hyaluronic acid (MeHA) synthesis

MeHA was synthesized following the procedure from Chung et al*.*^50^ with small modifications. Briefly 2 g hyaluronic acid (HA 75 kDa, Lifecore Biomedical) was dissolved in DI water at a concentration of 1 wt%. The solution was then cooled on ice and titrated with 5 M NaOH to a pH of 8.5. Next, 9.06 mL (0.05 mol) methacrylic anhydride (MA) was added in 750 µL doses, while titrating the solution back to a pH of 8.5 and waiting 5 min between additions. After the last dose of MA, the pH of the reaction was monitored and kept between pH 7.5–8.5 for 3 h by the addition of 5 M NaOH. The reaction was then removed from ice and allowed to proceed overnight. The next day the solution was transferred to dialysis tubing (6–8 kDa, Spectrum), and dialyzed against DI water for 7 days. The dialyzed solution was then frozen, lyophilized, and stored at – 20 °C until use. The MA functionalization was assessed via ^1^H NMR to be 22% (Fig. S1).

### Hydrogel formation

MeHA was dissolved in PBS (final concentration 4 wt%) with lithium phenyl-2,4,6-trimethylbenzoylphosphinate (LAP, 0.05 wt% final) and a cell-adhesive peptide (full sequence: GCGYGRGDSPG, 2 mM final). Next, 40 µL of this macromer solution was pipetted onto a Sigmacote (Sigma-Aldrich) treated glass slide. An 18 mm glass coverslip functionalized with (3-mercaptopropyl) trimethoxysilane was then placed on top, sandwiching the solution between the coverslip and the slide. The construct was then exposed to 365 nm light (10 mW/cm^2^, Omnicure S1500 lamp) for a predetermined amount of time, then left to sit for an additional 5 min for the gelation process to complete. The slide was then submerged in PBS, and the coverslip and bound hydrogel (referred to as hydrogel) was gently separated from the slide with a razor blade. The hydrogel was then allowed to swell in PBS at 37 °C overnight before cell seeding. PBS was replaced with full media 1 h before cell seeding to facilitate cell adhesion.

### Hydrogel stiffening

After 2 days of cell culture on hydrogel coverslips, media was aspirated and replaced with 0.05 wt% LAP in complete DMEM to further initiate crosslinking. Hydrogels were then incubated at 37 °C for 1 h to allow the LAP to diffuse evenly throughout the hydrogel. This media was then aspirated and replaced with 100 μL phenol red-free and serum-free media to minimize interference with 365 nm light penetration. The hydrogels were then exposed for 50 s to 365 nm light at an intensity of 10 mW/cm^2^. Immediately after stiffening, the phenol red-free media was replaced with complete DMEM.

### Rheological characterization of hydrogels

Dynamic shear moduli were measured on a TA Instruments HR-2 rheometer with an 8 mm parallel plate geometry. Hydrogel macromer solution (same formulation as in section “Hydrogel formation”) was placed on a quartz plate and exposed to 10 mW/cm^2^, 365 nm light in situ. A time sweep recorded the increase in the storage and loss moduli (G′ and G″, respectively) during the gelation process at 1 Hz and 1% strain. After gelation, a frequency sweep was performed from 0.01 to 100 Hz (Fig. S2) to confirm these measurements were in the linear viscoelastic range; all subsequent modulus measurements were then acquired at 1 Hz and 1% strain.

### Fluorescent microscopy and immunostaining

NHCFs on hydrogels were fixed following established procedures^26,51–53^ for 10 min at days 2, 4, and 6 with a 2% paraformaldehyde solution in PBS. After fixing, cells were permeabilized for 3 min with a 0.2% Trition X-100 and 2% paraformaldehyde solution in PBS. All experiments were performed with this same staining procedure; however, it is important to note that detection of α-SMA-positive cells may be underestimated with Triton X-100 permeabilization^54^. Next, the cells were blocked with 1% BSA blocking buffer for 1 h on a shaker table. After aspiration of the blocking buffer, 75 µL of primary antibody solution (Mouse anti α-SMA 3 µg/mL in blocking buffer (abcam cat# ab7817)) was placed at the bottom of a 12 well plate. The hydrogel was then flipped cell side down on top of the antibody solution, to ensure full coverage while minimizing the necessary volume, and stored at 4 °C overnight. Hydrogels were then flipped back cell side up and washed 3 × 5 min with PBS. Next, 75 µL of secondary antibody (Alexafluor 488 goat anti-mouse 1:200 (Invitrogen)) and rhodamine phalloidin (1:100 (Invitrogen)) were placed in the bottom of the well, and the hydrogels were flipped as for the primary antibody, and placed on a shaker table for 1 h. Hydrogels were then flipped cell side up and washed 3 × 5 min with PBS. Finally, the hydrogels were shaken with 500 µL of DAPI solution (1:1000 (Invitrogen)), followed by 2 final PBS washes. Hydrogels were then fixed on a glass slide using a gelvatol solution and allowed to dry overnight before imaging. All imaging was done on a Nikon Ti2-E eclipse microscope.

### Computation

The model was built using the TensorFlow python library^55^. The model training was carried-out on 4 NVIDIA Tesla V100 GPUs of the Longhorn supercomputer at the Texas Advanced Computing Center (TACC). Additionally training and test data sets are available on the Texas Data Repository at https://dataverse.tdl.org/dataverse/rosalesche.

### Statistical analysis

All error bars and boxes represent ± standard deviation. Results were analyzed with a one way ANOVA, and a two tailed student’s t test assuming equal variances. *P* values less than 0.05 were considered significant.

## Results

### Substrate elasticity effects on α-SMA stress fiber organization

**Figure 2:**
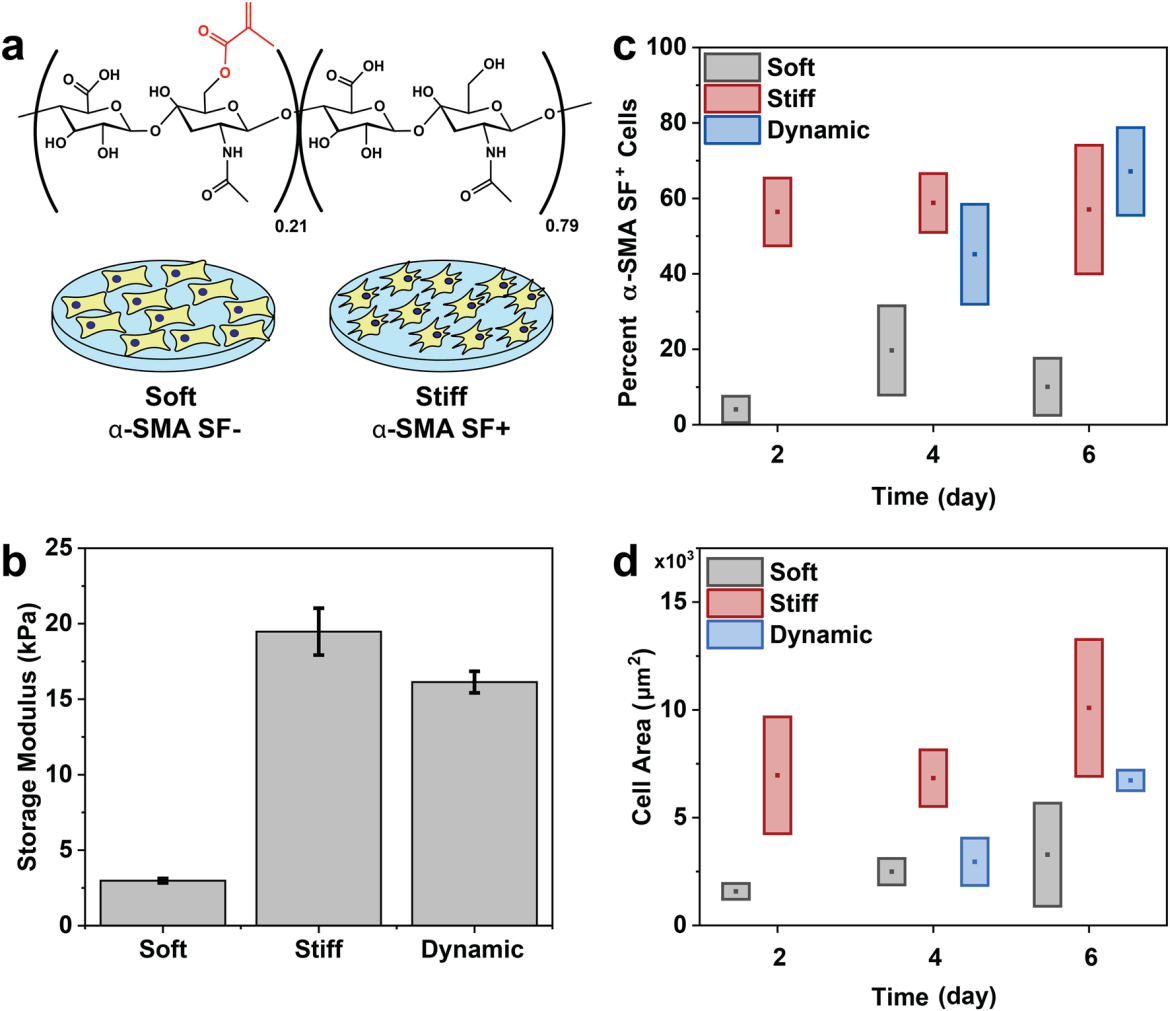
(**a**) Structure of methacrylated hyaluronic acid (MeHA) polymer for hydrogels. Cardiac fibroblasts were cultured on stiff substrates to promote α-SMA stress fiber organization. (**b**) Storage moduli for soft, stiff, and dynamic hydrogels as measured by shear oscillatory rheometry. (**c**) More cells display α-SMA stress fiber organization when cultured on stiff substrates, even when initial culture begins on soft substrates (as in the dynamic condition). (**d**) Cell area increases after at least two days of culture on stiff substrates. Boxes are ± 1 standard deviation; detailed statistics are shown in Tables S1 and S2.

To generate populations of cells with organized α-SMA stress fibers (α-SMA SF^+^), we cultured cardiac fibroblasts on MeHA hydrogels of various substrate elasticity (Fig. 2a). Based on previous reports^56^, a soft control (G′ = 3 kPa) was targeted to mimic the matrix stiffness of the healthy myocardium, and a stiff control (G′ = 20 kPa) was targeted to mimic end stage fibrotic cardiac tissue. To mimic the fibrotic progression from soft to stiff tissue, we also included a dynamic substrate that demonstrated rapid stiffening in situ after initial gelation via the photo-crosslinking of unreacted methacrylate groups. To facilitate cell attachment, the fibronectin-derived peptide RGD (Fig. S3) was incorporated into each hydrogel at the time of initial crosslinking and gelation.

To achieve the targeted moduli, the exposure time of photoinitiation (10 mW/cm^2^, 365 nm) was controlled: 10 s exposure for the soft condition and 300 s exposure for the stiff condition. The storage and loss moduli were measured using shear oscillatory rheology (Fig. 2b). After 2 days of cell culture, a portion of the soft hydrogels were exposed to an additional 50 s of 10 mW/cm^2^, 365 nm light, resulting in an increase in the storage modulus from 3 to 16 kPa (“Dynamic” condition, Fig. 2b). This additional dose of light was determined not to decrease cell metabolic activity (Fig. S4). Two, four, and six days after plating, cells were fixed, stained, and imaged in three channels, corresponding to distinct cellular components for counting and morphological analysis: (1) rhodamine phalloidin for F-actin, a part of the cytoskeleton that is common to both α-SMA SF^+^ and SF^−^ cells and illustrates cell shape, (2) DAPI for cell nuclei, to count number of cells and identify distinct cells, and (3) antibodies for α-SMA, for which organization into fibers is used to determine phenotype.

Cardiac fibroblasts were manually analyzed based on the presence of organized α-SMA stress fibers in individual cells (Fig. 2c). The presence of one stress fiber was considered sufficient to classify a cell as “α-SMA SF^+^”. Using manual counting methods, the cells cultured on the stiff substrates were found to be highly α-SMA SF^+^ over all 6 days (56% α-SMA SF^+^ D2, 59% α-SMA SF^+^ D4, 57% α-SMA SF^+^ D6), while cells cultured on the soft hydrogels remained relatively stress fiber free (4% α-SMA SF^+^ D2, 20% α-SMA SF^+^ D4, 10% α-SMA SF^+^ D6). For the dynamic condition, it was observed that stress fiber formation increased tenfold (4% → 45%) only 2 days after hydrogel stiffening. Furthermore, at 4 days post-stiffening, the cell population on the dynamic hydrogel matched the α-SMA SF^+^ level of the cell population cultured on the static, stiff control (67% α-SMA SF^+^ dynamic D6). Stress fiber organization did not significantly correlate with relative expression of alpha-smooth muscle actin (ACTA2) measured for the same conditions (Fig. S5).

Average cell size, which has been shown to correlate with myofibroblast activation^57^ and stress fiber formation, was also measured (Fig. 2d). In accordance with the α-SMA fiber analysis, cells on the static stiff substrate were significantly larger (5400 μm^2^ D2, 6700 μm^2^ D4, 8700 μm^2^ D6) than those on the static soft substrate (1300 μm^2^ D2, 1300 μm^2^ D4, 1400 μm^2^ D6) across all 6 days. While the levels of α-SMA SF^+^ cells remained relatively constant over 6 days, the cells cultured on the stiff substrate exhibited an increase in average cell area over time. Two days after stiffening, cells cultured on the dynamic substrate had roughly doubled in size (1300 μm^2^ D2, 3000 μm^2^ D4) and doubled in size again 4 days post-stiffening (6300 μm^2^ D6).

In total, this experiment generated 306 images (containing 2942 individual cells) with images where 0–100% of the cells displayed stress fiber organization, depending on the elasticity of the underlying substrate. To reduce the time burden of manual classification, as well as to reduce variation between researchers, we next created a computational model that could automate stress fiber identification in a shorter time.

### Model design

**Figure 3:**
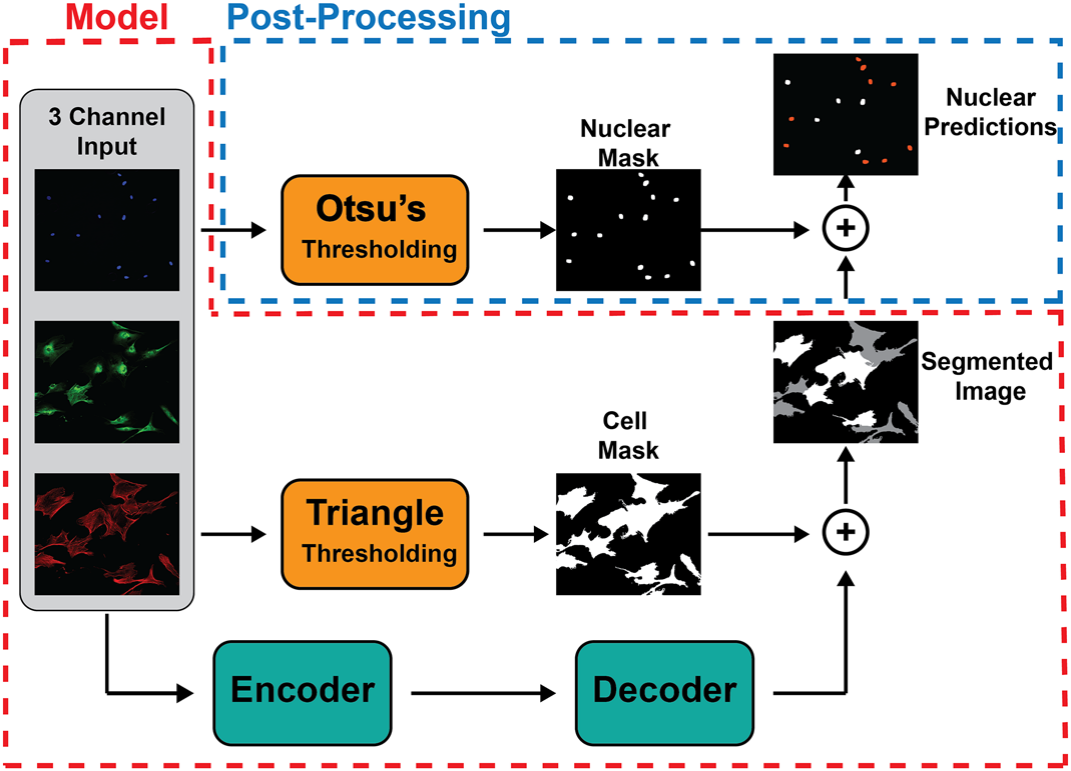
General workflow. The model supplements an encoder-decoder deep learning architecture with binary thresholding to generate a segmentation map. Post-processing then applies a nuclear segmentation map to predict individual cell phenotype.

We developed a model that first applies traditional image processing methods, such as thresholding, to simplify the problem, then uses deep learning to achieve segmentation (Fig. 3). In contrast with traditional methods, deep learning models are comprised of a series of layers with tunable parameters. For instance, in the case of semantic segmentation, the model receives an input image and passes it through these layers to generate an output prediction. The parameters within each layer are then optimized to minimize the mismatch between this prediction and a ground truth (expert labeled image). These models can be made using many different architectures, each optimized for a specific task. Some of the most well established deep learning models for semantic segmentation utilize an encoder/decoder architecture (Fig. S6), where an encoder first downscales the image to distill the most essential features for the given task (segmentation), and then a decoder upscales this representation to build a segmented version of the input image. Each of these components is comprised of a series of residual blocks^58^. Residual blocks allow for the training of models containing many layers, which in turn, allows the model to capture more complex relationships. The encoder and decoder are connected by a series of paths at different depths^39^, which allow for the flow of information between different scales. These connections help model training while also preserving semantic information from the input and passing it directly to the decoder. Combined, these components have previously been applied to a variety of segmentation tasks including biological applications, such as cell segmentation^39,59^.

**Figure 4:**
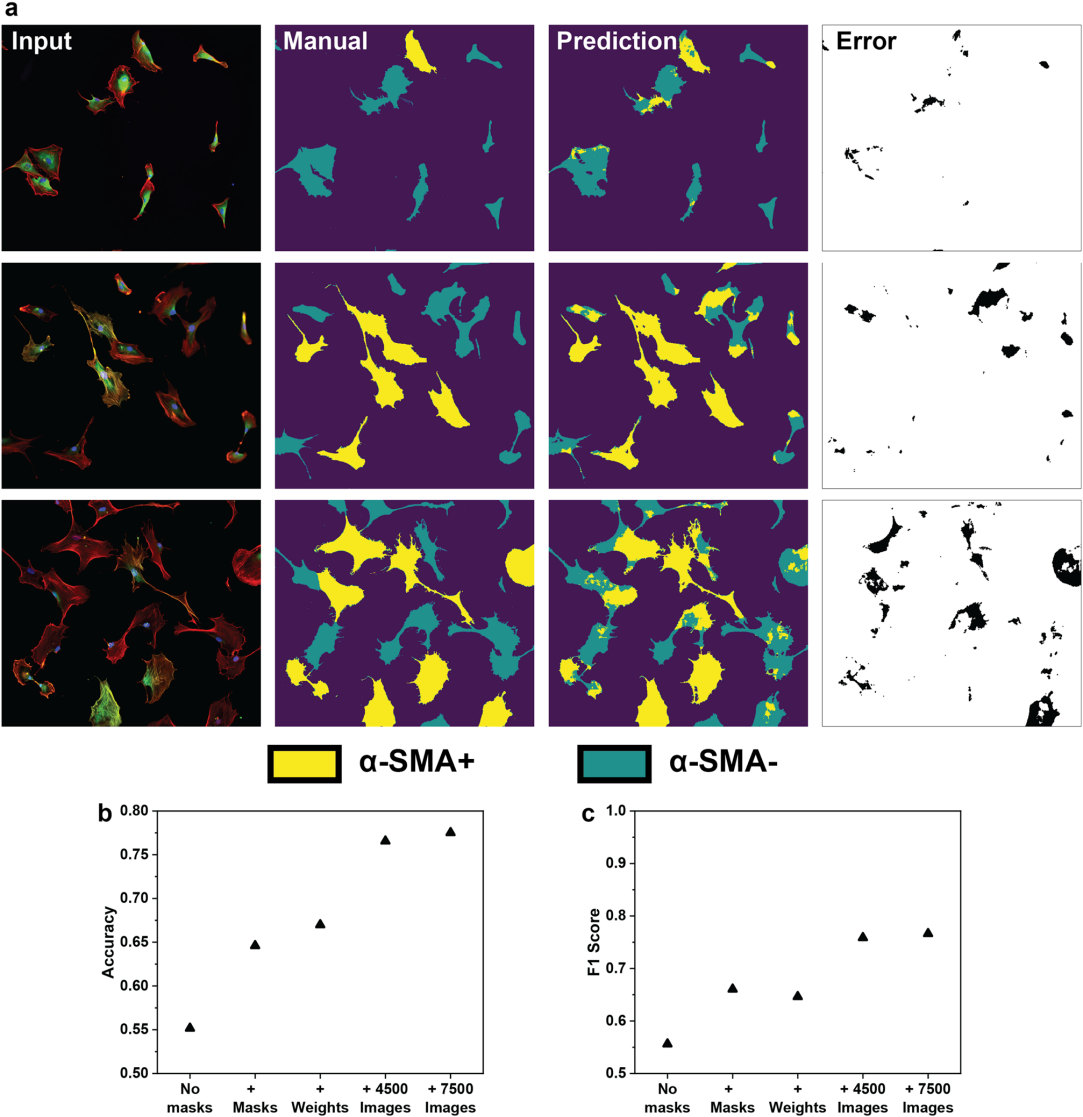
Sample results of 3 images from the test set. (**a**) The first column shows the fluorescent input images, the second column shows cells with organized α-SMA stress fibers as determined by manual identification, the third column shows the model prediction, and the final column highlights areas in the image where the model prediction deviates from the manual identification. (**b**) Model accuracy increased with the addition of class weights and data augmentation. (**c**) The false positive/false negative (FP/FN) ratio was used to measure model bias towards a particular class.

Given the aforementioned 3 channel input, our goal was to semantically segment, or label each pixel within an image, as one of 3 classes: α-SMA SF^+^ cell, α-SMA SF^−^ cell, or background. We first employed an established encoder-decoder model architecture similar to those previously reported^39^. This model struggled to differentiate any of the 3 classes; it had an accuracy of 55%, and a mean IOU of 11% (Figs. 4 and S8, “No masks” model). To improve performance, we next employed a customized computer vision model. The first layer of the model applies the triangle thresholding algorithm^60^ and a minimum size cutoff of 300 μm^2^ (to remove cellular debris) to the F-actin channel only, in order to segment cells from the background. This process is highly efficient because of the high contrast provided by the fluorescent phalloidin stains. This cell mask is then passed directly to the decoder, where it is incorporated before the final activation layer. This method of masking the background allows the model’s parameters to be trained exclusively to label the two cell-types since the background pixels will not produce a training signal. Next, the 3 channel image is passed to the encoder, which is made up 4 residual blocks, each downscaling the information by a factor of 2. The information is then passed to the decoder which upscales it into a segmentation map with the same dimensions as the original input. By taking advantage of traditional image processing techniques (such as thresholding) and combining them with standard deep learning components, we were able to construct a model capable of segmenting α-SMA SF^+^ cells.

### Model training and evaluation

Our model was trained on 300 unique images (note, these are 300 new images, different than those referenced in section “Substrate elasticity effects on α-SMA stress fiber organization”, see Table S3), containing between 5 and 30 cells each, collected over the course of 7 different cardiac fibroblast activation experiments on substrates of various moduli. Images were originally captured at high resolution (2160 × 2560), then scaled to 560 × 640 for model training. The scaling minimized the memory needed to train the model, while maintaining a high enough resolution to identify activated cells manually. While training each iteration of the model, the training set was randomly split such that 80% of the images were used for model training and 20% of the images were used for validation after each training epoch. Tables S4 and S5 have more information about model size and specific hyperparameters used for training.

In order to evaluate the pixel-wise accuracy of the model, we created a test set of 20 images (195 total cells of which 45% were manually classified as α-SMA SF^+^). These images were collected during the same experiments as the training set images; however, they were withheld during the training process and used to compare the performance of different model iterations. They encompassed a range from relatively simple single cells to more complex clusters of cells with multiple cell–cell contacts (select images from test set Fig. S7). Many of the cells themselves contained intermediate phenotypic markers such as few stress fibers, heterogeneity in α-SMA staining within the cell, extended cell shape, or dim staining. The model output classifies each pixel in the image as “α-SMA SF^+^,” “α-SMA SF^−^,” or “background” (Fig. 4a).

A significant difficulty in evaluating the model was the translation of the segmentation mask (individual pixel predictions) into a count of α-SMA SF^+^ cells. Because cells are often clustered together, it becomes very difficult to determine clear boundaries between individual cells. In order to determine the number of cells and their location within the image, we assumed that each nucleus corresponds to a single cell. We found that a generalized Otsu’s thresholding algorithm^61^ worked best to segment cell nuclei from the DAPI channel, because of its ability to handle greater variations in the image intensities. This algorithm works well due to the consistently large contrast associated with DAPI staining; however, it is important to note that changes in the calculated threshold value could slightly change the shape of the segmented nucleus and therefore have an effect on the final model output. We combined this algorithm with a size cutoff range of 100–3000 μm^2^ to accurately segment only the cell nuclei. The segmentation mask from the DAPI channel was then superimposed on the model output, and the average class (or predicted phenotype) of each nuclear region was used to determine the phenotype of each cell. Importantly, Otsu’s thresholding was only applied to the DAPI input channel to identify a “nuclear region of interest;” the model output of this region remained unchanged (Fig. 3). Using this method, the model was trained on the 300 image training set, and an accuracy of 65% was achieved (Fig. 4a), i.e. 65% of cells were assigned the same label by both the manual analysis and the model. Interestingly, the model appeared to over-predict the α-SMA SF^+^ class, as quantified by a false positive to false negative ratio (FP/FN) of 1.85 on the test set (Fig. 4b). This is likely due to a class imbalance (2.5:1) towards α-SMA SF^+^ cells within the training set. In order to correct this imbalance, we applied weights to each sample so that images containing a greater fraction of α-SMA SF^-^ cells were given a higher weight in the loss function. This method slightly increased the accuracy to 67% and decreased the FP/FN to 0.97 (Fig. 4b).

Given the small number of unique images in the training set, we explored a variety of methods to further increase model accuracy. The first method, data augmentation, took the original images and randomly zoomed and cropped, sheared, flipped, or dimmed/brightened them to generate 4800 images. Retraining the model on this expanded training set resulted in an increase in accuracy up to 77%, and an F1 score of 0.76. Further expansion of the training set to 7800 images resulted in an increase to 78.5% accuracy and an F1 score of 0.77 (Fig. 4b, c), and additional augmentation did not see a further increase in accuracy. For this final model, the training IOU for α-SMA SF^-^ was 0.654, while the validation IOU was 0.644. This shows that the model was not significantly overfitted. We also trained a model to directly predict the class of only the nuclear area; however, this model had significantly lower accuracy than the model trained to predict full cells. We hypothesize that this is due to limitations caused by the structure of the cell. It was often observed that the nuclear region had lower levels of a-SMA present than cytoplasmic regions, resulting in a lesser phenotypic difference between α-SMA SF^+^ and α-SMA SF^−^ cells. By applying nuclear masks in post-processing rather than within the model, the model is able to use the entire cell to make its prediction, increasing its accuracy. Lastly, we incorporated additional layers such as attention^62^, squeeze and excite^63^, and pyramidal pooling^64^. However, none of these increased model accuracy further. We hypothesize that this is due to limitations of the small amount of training data (Fig. S9 shows model metrics improving significantly as number of training images increases).

### Model application

**Figure 5:**
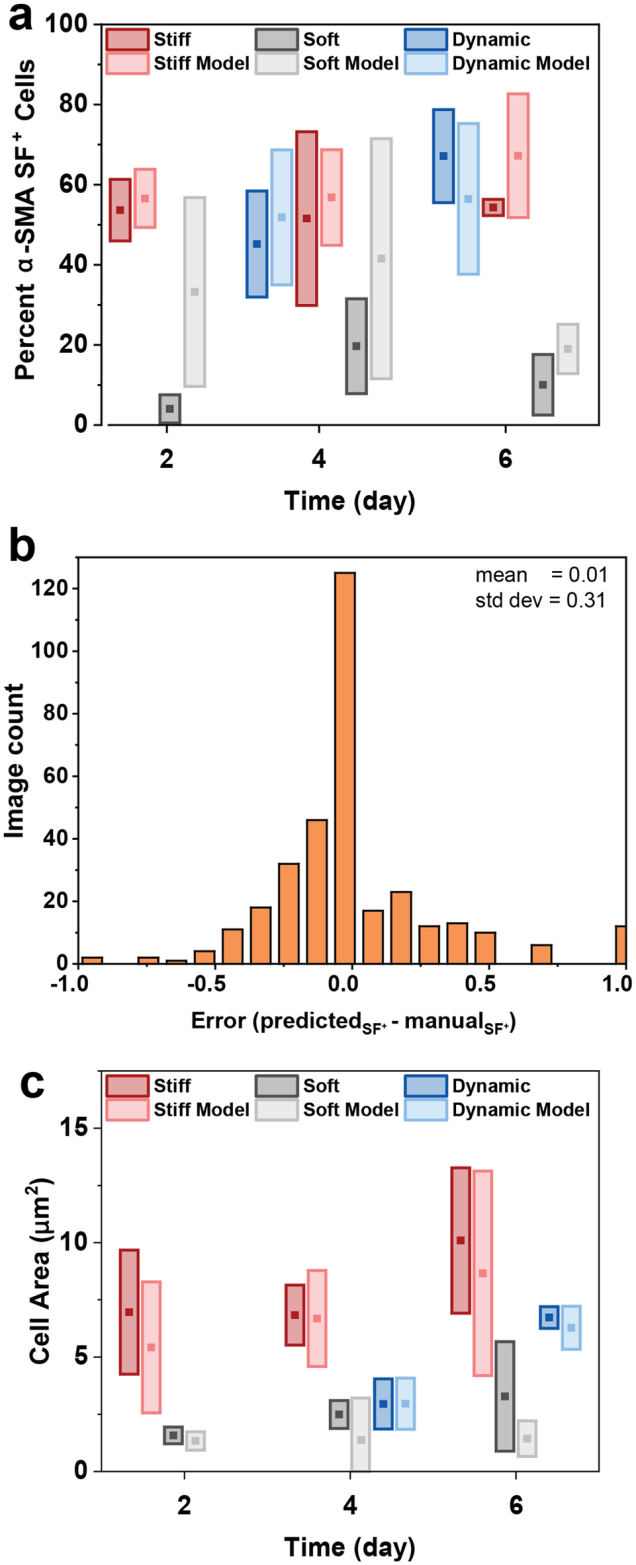
(**a**) Percentage of α-SMA SF^+^ cells predicted by the model (light bars) and manual identification (dark bars). (**b**) Visualization of the difference between model and manual identification on an individual image scale. For 98/306 images, the results matched exactly. (**c**) Comparison of average cell area predicted by the model (light bars) and manual analysis (dark bars). Detailed statistics are shown in Tables S1 and S2.

To demonstrate the effectiveness of our model, we applied it to the dataset collected in Fig. 2 and compared the results to the manual analysis (Fig. 5a). During model development, the training data was normalized so that the mean intensity of each channel was 0. This same normalization worked well for the test set because the images were originally from the same larger set of experiments. However, the substrate stiffening dataset (Fig. 2d) was collected at a later time, under different microscope settings. As a result, the mean intensities of each channel were significantly different than those of the training set. In order to correct for this, the experimental images were normalized by a different set of values than the training set. This normalization was determined by selecting a small subsample of application images and optimizing these normalization parameters until the model best matched the manual analysis. It is expected that this normalization will need to be adjusted, as a type of calibration, when processing images from a different microscope or taken with different settings.

The model predicted fibroblasts cultured on the stiff substrate to be 49% α-SMA SF^+^ on day 2, 52% α-SMA SF^+^ on day 4, and 54% α-SMA SF^+^ on day 6. It also predicted fibroblasts cultured on the soft substrate to be 36% α-SMA SF^+^ on day 2, 41% α-SMA SF^+^ on day 4, and 19% α-SMA SF^+^ on day 6. For the dynamic condition, the model predicted 52% α-SMA SF^+^ fibroblasts on day 4 and 57% α-SMA SF^+^ on day 6. Although the model over-predicted the amount of α-SMA SF^+^ fibroblasts cultured on the soft substrate compared to the manual analysis, it captured the trend of increasing stress fiber organization on stiffer substrates compared to soft substrates. Furthermore, to analyze the model performance by image, we calculated the difference between the predicted and manual values (Fig. 5b). In this analysis, positive values correspond to an over prediction of α-SMA SF^+^ levels, while negative values correspond to an under prediction. Finally, we measured average cell area using our model by dividing the F-actin segmentation mask by the number of nuclei in each image. For this measure, the model matched the manual analysis for all substrate conditions (Fig. 5c).

## Discussion and conclusion

We have developed a computer vision model that is able to segment cells containing α-SMA stress fibers from a standard, 3 channel fluorescence image. This model was applied to a set of images containing cardiac fibroblasts with a range of α-SMA expression levels and organization states. We specifically controlled α-SMA expression levels by culturing cardiac fibroblasts on soft, stiff, or dynamic hydrogel substrates to mimic the mechanical changes in extracellular matrix during fibrotic progression. Our model captured the same trends in stress fiber organization as reported using a manual classification analysis, and its predictions were statistically the same as the manual analysis for all conditions. For the soft condition, our model over predicted the amount of α-SMA SF^+^ cells, although the prediction is still significantly lower than that of the stiff and dynamic conditions on all days.

Automating the identification of α-SMA stress fiber assembly is a complex computer vision task of relevance to many biological contexts. Here, our use of a mechanically tunable hydrogel model with a mechanism for in situ control highlights the rapid development of biomaterials platforms that can screen numerous cell–matrix conditions. While we explored the effect of increasing mechanics on fibroblast activation at one timepoint, one could easily envision generating data from additional stiffening timepoints, additional moduli besides 3 kPa or 20 kPa, or additional culture times or cell densities. Toward this end, many hydrogel platforms are under development for incorporation into high throughput screening platforms that utilize small volumes and rapid liquid handlers^65^. These platforms have the potential to generate large amounts of image-based datasets, which further highlights the need for automated data processing methods that can predict cell phenotype.

While our model meets a need for automated stress fiber identification, it is important to note a few limitations of this model. Firstly, because of the relatively small amount of training data, our model requires an empirical calibration before application to new data sets, which corrects for small experimental variations such as differences in fluorescent stain intensity and microscope settings. This limitation could be addressed in the future by retraining the model with a larger and more diverse dataset, containing images with different staining procedures and taken under different microscope settings. Secondly, due to the difficulty of segmenting individual cells in fluorescent images, our post-processing procedure only considers the predicted class of the nuclear region of each cell. Segmentation of 2D fluorescent images is of future interest to the authors, and once successful, will enable the model to use the predicted class of the entire cell in its output. Lastly, because all labels were manually created by a single researcher, this model contains an inherent bias. This limitation could be addressed in the future either by developing a “consensus dataset” that combines the opinions of multiple researchers in the field, or by using unsupervised learning to remove the need for manually-assigned labels entirely.

Due to the commonality of α-SMA stress fibers between myofibroblasts of different tissues, this model has the potential to be applied towards identifying phenotypes in cells from many sources. As with all neural networks, we are limited by the training data available. Future iterations of this model would be improved through inclusion of more cardiac fibroblast images, as well as images of other fibroblast types.

## Supplementary Information

Supplementary Information.

## Data Availability

All code is available with test set images at https://github.com/ahillsley/cellnet. Training and test data is also available on the Texas Data Repository at https://dataverse.tdl.org/dataverse/rosalesche. All other data is available upon request.
