# Supplementary material for: A deep learning approach to identify and segment alpha-smooth muscle actin stress fiber positive cells
Source: Sci Rep. 2021 Nov 8;11:21855. doi: 10.1038/s41598-021-01304-4 (PMC8575943; doi:10.1038/s41598-021-01304-4)
Supplement: Supplementary file 1 — Supplementary Information. [file 41598_2021_1304_MOESM1_ESM.pdf]

Supplementary Information

A Deep Learning Approach to Identify and Segment alpha-Smooth Muscle Actin Stress Fiber Positive Cells

Alex Hillsley[1], Javier E. Santos[2], Adrianne Rosales[1,*]

[1] McKetta Department of Chemical Engineering, University of Texas at Austin, Austin, TX USA

[2] Hildebrand Department of Petroleum and Geosystems Engineering, University of Texas at Austin, Austin TX USA

* Email address: arosales@che.utexas.edu; Tel: +1 512-471-6300

Supplementary methods:

Peptide Synthesis

A cell adhesive peptide (GCGYGRGDSPG) was synthesized using Rink amide polystyrene resin (0.25 mmol scale, 0.72 mmol/g, Chem-Impex) on a Prelude X automated peptide synthesizer (Gyros Protein Technologies). After synthesis, the peptide was cleaved from the resin for 4 hours with 15 mL of cleavage solution (95: 2.5: 2.5, trifluroacetic acid: water: triisopropylsilane). The peptide was then purified via high pressure liquid chromatography (HPLC, Dionex UltiMater 3000, C18 column) using a 25 min gradient of water and acetonitrile (10-30% acetonitrile). The desired mass was confirmed using MALDI-TOF (Figure S3), and the peptide was lyophilized and stored at -20 ºC until use.

Coverslip functionalization

To functionalize coverslips with a methacrylate group for covalent hydrogel attachment, 18 mm round glass coverslips (ChemGlass) were first submerged in a 10M NaOH solution and placed on a shaker table for 1 hour. Each coverslip was then individually rinsed 3x with DI water and dried under compressed air. Coverslips were then submerged in the functionalization solution (0.4% 3-(Trimethoxysilyl)propyl methacrylate in acetone) at room temperature for 20 min. Each coverslip was then individually washed once with acetone and placed in a glass dish to dry.

RT-qPCR

For all PCR experiments, cell seeding density was increased to 5,000 cells/cm^2 in order to increase the RNA yield per hydrogel; additionally, RNA from 6 hydrogels was combined into each replicate. RNA was isolated from attached cells using an RNeasy mini kit (Qiagen). Amount of RNA was quantified, and purity measured on a Nanodrop One (Thermo Scientific). RNA was then reverse transcribed into cDNA using a Veriti thermocycler (Applied Biosystems). ΔΔCt qPCR was then run on a Quantstudio 3 (Applied Biosystems), using SYBR green reagents and 3 ng cDNA per 20 µL reaction. ACTA2 primer (Integrated DNA Technologies) sequence, FW: GACAATGGCTCTGGGCTCTGTAA, RV: CTGTGCTTCGTCACCCACGTA.

Antibody information

Primary mouse monoclonal antibody to alpha smooth muscle actin was purchased from abcam (Clone: 1A4, Subtype: IgG2a, Catalog number ab7817, Lot No: GR3190124-5)

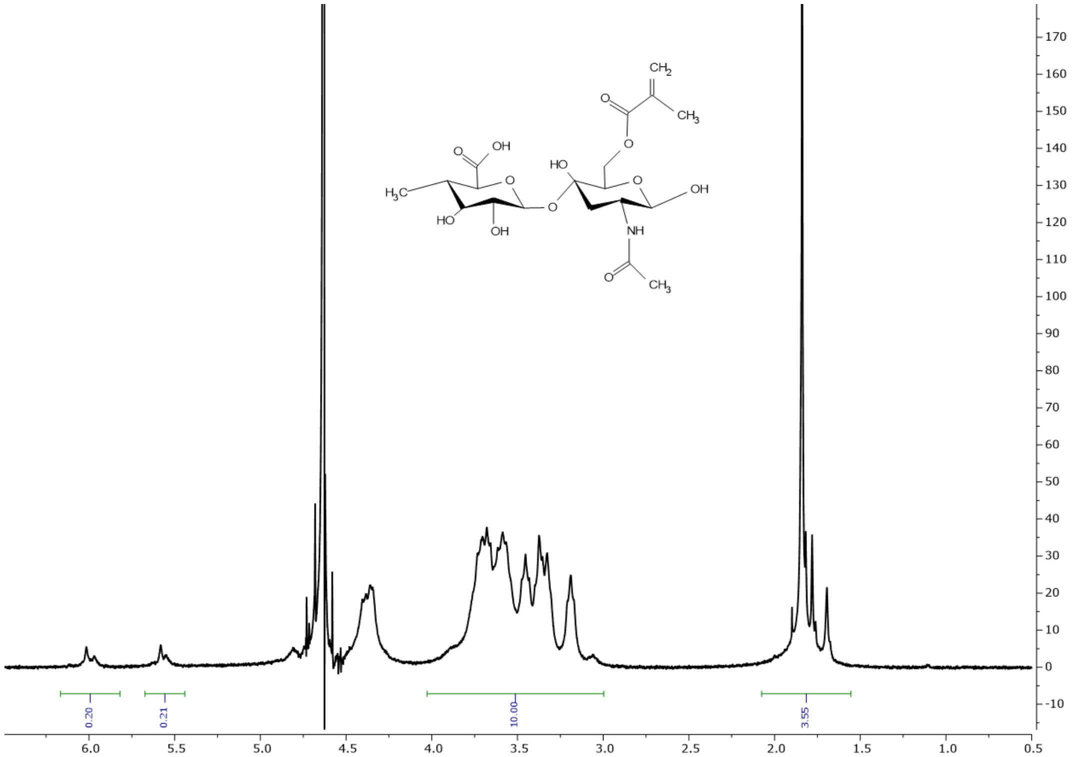

Figure S1: NMR of Methacrylated HA (MeHA) in D_2O. It was determined that ~20% of repeat units were functionalized with a methacrylate group by normalizing the backbone peaks (3.0 – 4.0) to 10 and measuring the alkene peaks at 5.1 and 6.0.

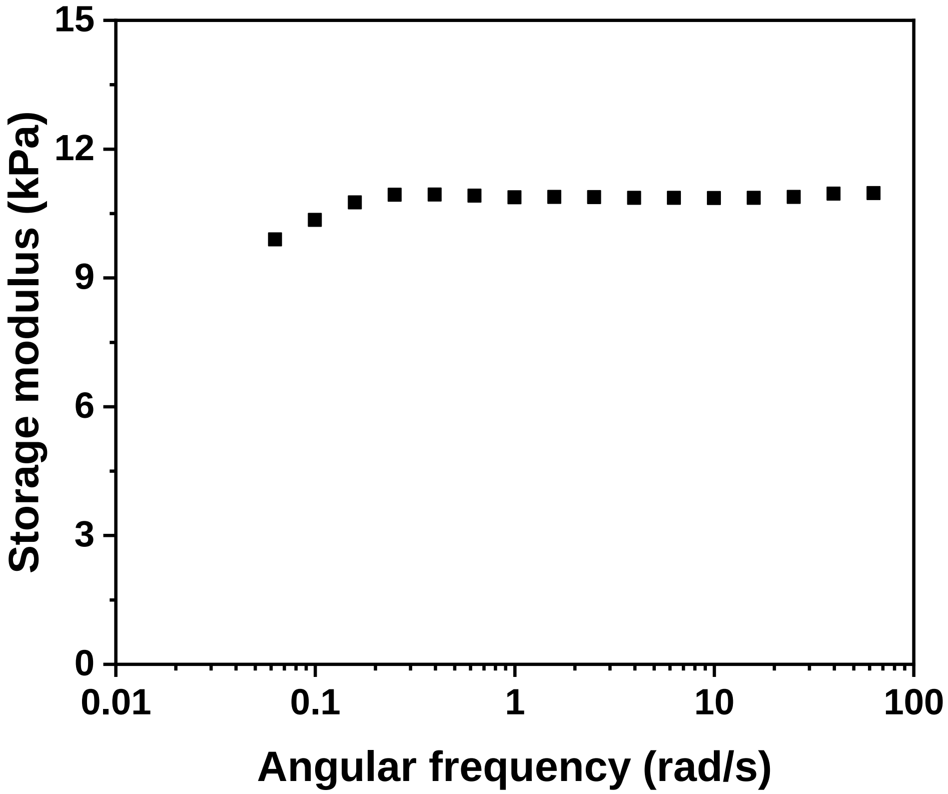

Figure S2: A frequency sweep of a pre-stiffened MeHA hydrogel. The modulus was found to be independent of frequency, which indicates an elastic network. All further rheological measurements were taken at a frequency of 1 rad/s.

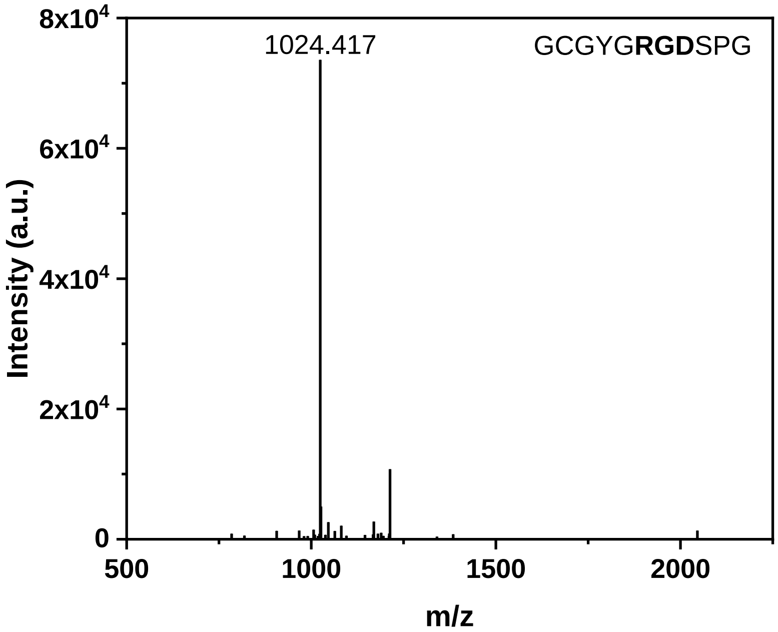

Figure S3: MALDI-TOF trace of HPLC purified RGD peptide, expected m/z is 1024.

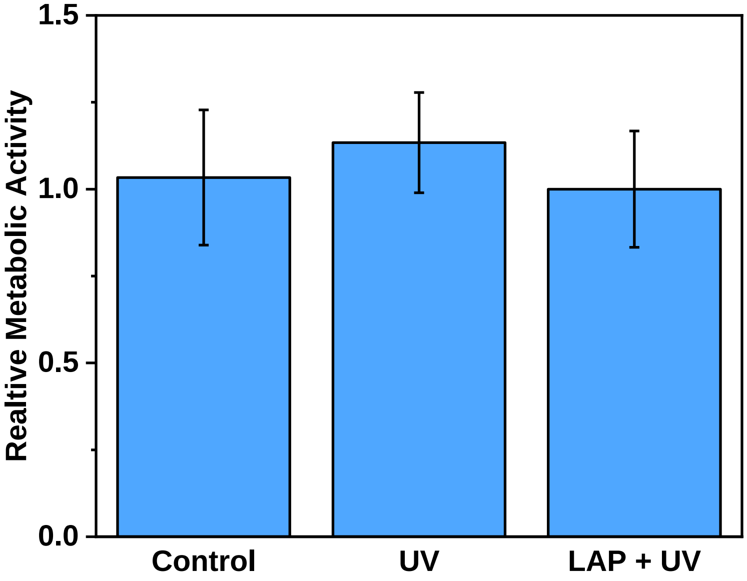

Figure S4: After 2 days in culture, cells were incubated with 0.05 wt% LAP for 1 hour, then exposed to 50s of 10 mW/cm^2 UV light. Cells were then cultured an additional 2 days, and metabolic activity was measured using a Vybrant MTT Cell proliferation Assay. LAP incubation and short UV exposure were found to have no significant effect on cell metabolic activity.

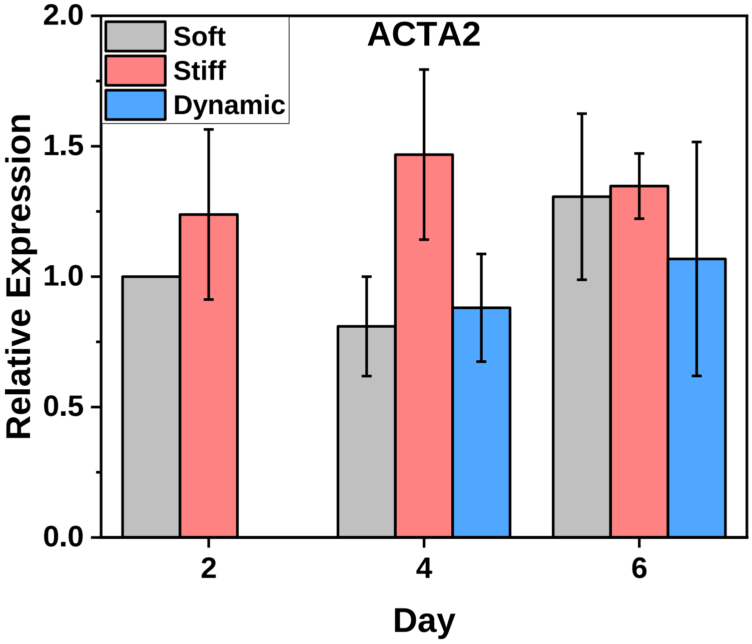

Figure S5: Gene expression of alpha smooth muscle actin (ACTA2) was measured for each condition (Soft / Stiff / Dynamic, on days 2, 4, and 6). No statistically significant differences in expression were observed, although there is a slight increase in ACTA2 expression on stiff substrates at days 2 and 4.

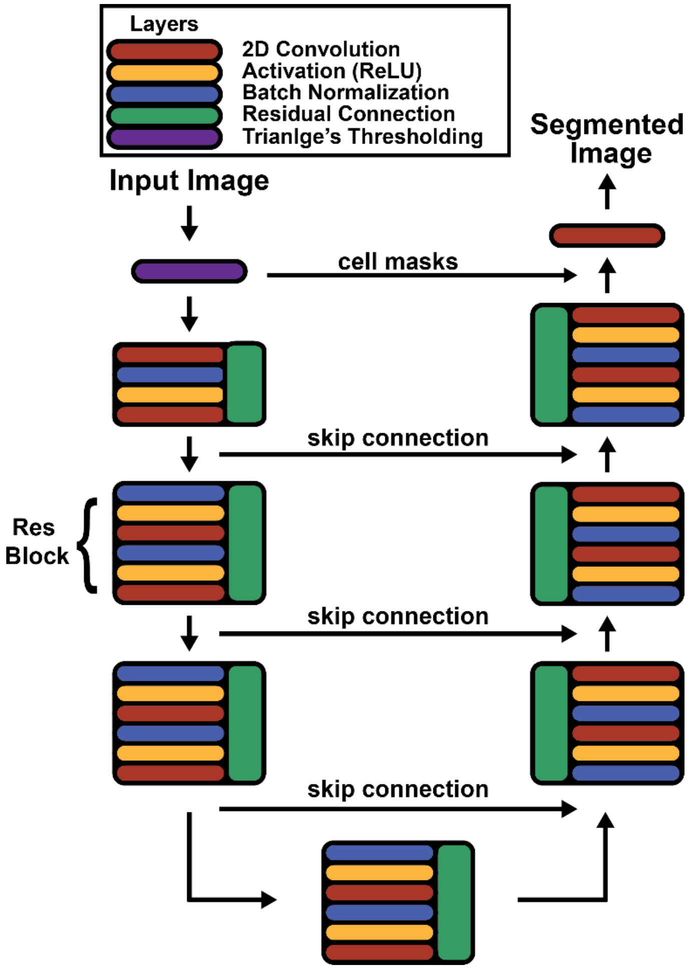

Figure S6: Diagram of the encoder-decoder structure of our model. Importantly, our architecture, creates binary cell masks from the input and passes them directly to the top of the decoder. This allows models power to focus on differentiating between the two cell phenotypes.

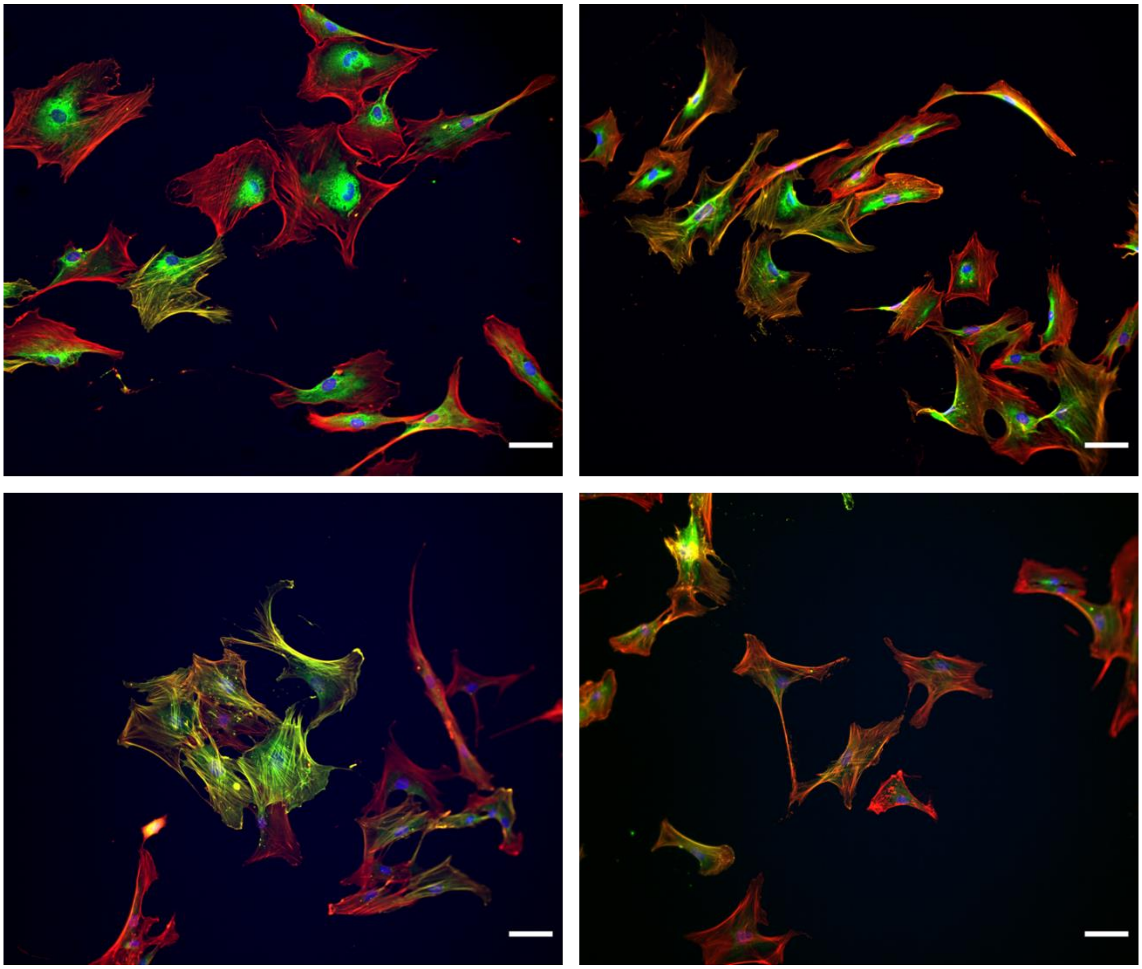

Figure S7: Representative 3-channel fluorescent images from the 20 image test set. Red = F-actin, Green = alpha-smooth muscle actin, Blue = Nucleus, scale bar = 33 μm.

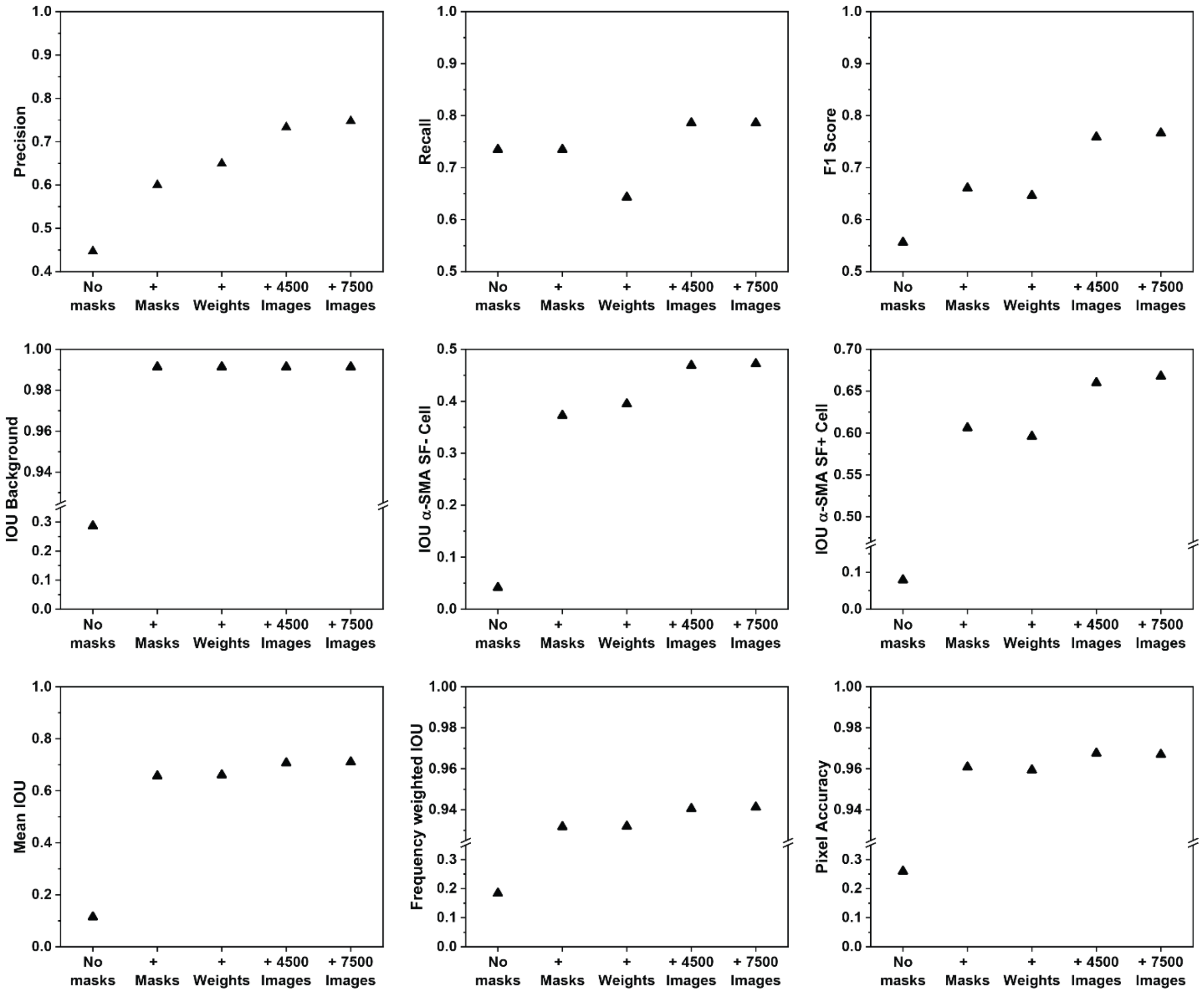

Figure S8: Model metrics from the test set of 20 images, comparing the performance of different model iterations. Metrics include: Precision, Recall, F1 score, IOU for each of the 3 classes, Mean IOU, Frequency weighted IOU, and total Pixel Accuracy.

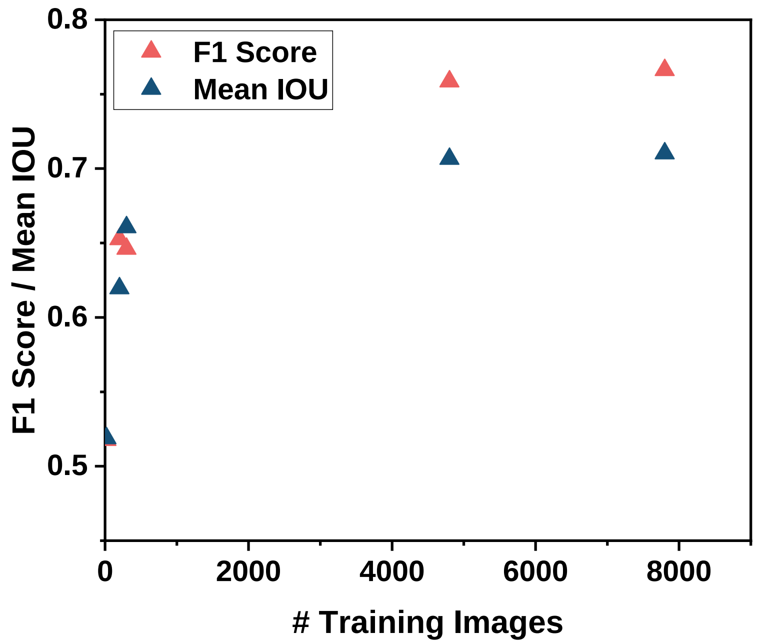

Figure S9: Our best models (containing both masks and image weights) were re-trained on image sets containing 20, 200, 300, 4,800, and 7,800 images. Importantly the 4,800 and 7,800 image sets contain only 300 unique images with the rest being generated through augmentation.

A

	Manual Soft / Manual Stiff	Manual Stiff / Manual Change	Manual Soft / Manual Change	Model Soft / Manual Soft	Model Stiff / Manual Stiff	Model Change / Manual Change	Model Soft / Model Stiff	Model Stiff / Model Change	Model Soft / Model Change
**D2**	0.001	-	-	0.101	0.708	-	0.227	-	-
**D4**	0.009	0.200	0.068	0.305	0.615	0.618	0.664	0.985	0.631
**D6**	0.012	0.444	0.002	0.187	0.798	0.450	0.001	0.854	0.031

B

Manual Soft	0.147
Manual Stiff	0.969
Manual Change	0.097
Model Soft	0.499
Model Stiff	0.965
Model Change	0.768

Table S1: Statistical analysis of α-SMA SF^{+} cell experiments, with data shown in Figures 2C (only manual analysis) and 5A (including model predictions). Conditions that are statistically significant ($P < 0.05$) are highlighted. A) Results of a two tailed student's t-test assuming equal variances. B) Results of a one way ANOVA across all time points for each experimental condition.

A

	Manual Soft / Manual Stiff	Manual Stiff / Manual Change	Manual Soft / Manual Change	Model Soft / Manual Soft	Model Stiff / Manual Stiff	Model Change / Manual Change	Model Soft / Model Stiff	Model Stiff / Model Change	Model Soft / Model Change
**D2**	0.027	-	-	0.480	0.536	-	0.071	-	-
**D4**	0.007	0.012	0.071	0.363	0.923	0.511	0.029	0.054	0.262
**D6**	0.041	0.357	0.079	0.272	0.673	0.359	0.051	0.418	0.002

B

Manual Soft	0.406
Manual Stiff	0.277
Manual Change	0.031
Model Soft	0.994
Model Stiff	0.518
Model Change	0.017

Table S2: Statistical analysis of cell size measurements, with data shown in Figures 2D (only manual analysis) and 5C (including model predictions). Conditions that are statistically significant ($P < 0.05$) are highlighted. A) Results of a two tailed student's t-test assuming equal variances. B) Results of a one way ANOVA across all time points for each experimental condition.

Image Sets	# Images	Comments
**Training**	300	• Split 80% training / 20% Validation
**Test**	20	• Used to evaluate model preformance<br>• Not seen during training<br>• Used in Fig 4 and S7
**Application**	306	• Images from an independent cell experiment<br>• Not seen during training<br>• Used in Fig. 2 and 5

Table S3: Details of the 3 different image sets used throughout this manuscript.

Block	# filters
Encoder_1	24
Encoder_2	48
Encoder_3	96
Bridge	192
Decoder_3	96
Decoder_2	48
Decoder_1	24

Table S4. Specific model details including the number of convolutional filters in each ResBlock within the encoder and decoder.

Model Hyperparameters	
Batch Size	32
Learning Rate (LR)	1.00E-05
Training validation split	80/20
Reduce LR after _ epochs	50
Kernel size	3 x 3

Table S5. Summary of model hyperparameters.
